# A cost-effective technique for generating preservable biomass smoke extract and measuring its effect on cell receptor expression in human bronchial epithelial cells

**DOI:** 10.1093/biomethods/bpy010

**Published:** 2018-10-04

**Authors:** K C Rajendra, Graeme R Zosky, Shakti D Shukla, Ronan F O’Toole

**Affiliations:** 1 College of Health and Medicine, University of Tasmania, Hobart, Tasmania, Australia; 2 School of Biomedical Sciences and Pharmacy, University of Newcastle, Callaghan, New South Wales, Australia

**Keywords:** biomass smoke, cigarette smoke extract, chronic obstructive pulmonary disease, platelet-activating factor receptor

## Abstract

Nearly half of the world’s population uses biomass fuel for the purposes of cooking and heating. Smoke derived from biomass increases the risk of the development of lung diseases, including pneumonia, chronic obstructive pulmonary disease, airway tract infections, and lung cancer. Despite the evidence linking biomass smoke exposure to pulmonary disease, only a small number of experimental studies have been conducted on the impact of biomass smoke on airway epithelial cells. This is in part due to the lack of a standard and easily accessible procedure for the preparation of biomass smoke. Here, we describe a cost-effective and reproducible method for the generation of different smoke extracts, in particular, cow dung smoke extract (CDSE) and wood smoke extract (WSE) for use in a range of biological applications. We examined the effect of the biomass smoke extracts on human bronchial epithelial cell expression of a known responder to cigarette smoke exposure (CSE), the platelet-activating factor receptor (PAFR). Similar to the treatment with CSE, we observed a dose-dependent increase in PAFR expression on human airway epithelial cells that were exposed to CDSE and WSE. This method provides biomass smoke in a re-usable form for cell and molecular bioscience studies on the pathogenesis of chronic lung disease.

## Introduction

It is estimated that nearly 3 billion people worldwide are exposed to biomass smoke, generated from burning wood, crop residues, or animal dung for household cooking and heating [1]. Biomass smoke is the leading environmental cause of death and disability, causing over 4 million deaths each year [2]. Several epidemiological studies have associated biomass smoke exposure with lung diseases, including chronic obstructive pulmonary disease (COPD), airway infections, and lung cancer [3–7]. Similarly, *in vitro* studies have found that human lung cells exhibit impaired inflammatory and immune responses following exposure to biomass smoke [8, 9]. Inhalation of animal dung biomass smoke is of particular concern to human health as it has the highest polluting potential per unit energy released compared to wood smoke [10]. Airway epithelial cells are the primary target of inhaled smoke; therefore, the responses of epithelial cells to different types of biomass smoke are of considerable interest. Although, extensive *in vitro* studies have been performed on the effects of tobacco smoke on the expression of host receptors on respiratory epithelial cells and on susceptibility to bacterial infection [11–13], only a small number of comparable studies have been performed using biomass smoke. More mechanistic research is therefore needed to understand the cellular and molecular responses to biomass smoke, including animal dung and wood smoke. Currently, we do not have standardized experimental approaches for the preparation of re-usable biomass smoke extract and for the assessment of cellular responses to different types of biomass smoke. Here, we devised a low-cost and reproducible biomass smoke generation system and tested the extracts for their effect on human bronchial epithelial cell expression of platelet-activating factor receptor (PAFR), a G-protein-coupled receptor (GPCR), and an established marker of cigarette smoke exposure [13–15]. GPCRs constitute a large family of membrane-bound receptors that activate intracellular signal transduction pathways in eukaryotic cells in response to extracellular signals [16].

## Materials and methods

### Preparation of cigarette smoke extract

**Figure 1:**
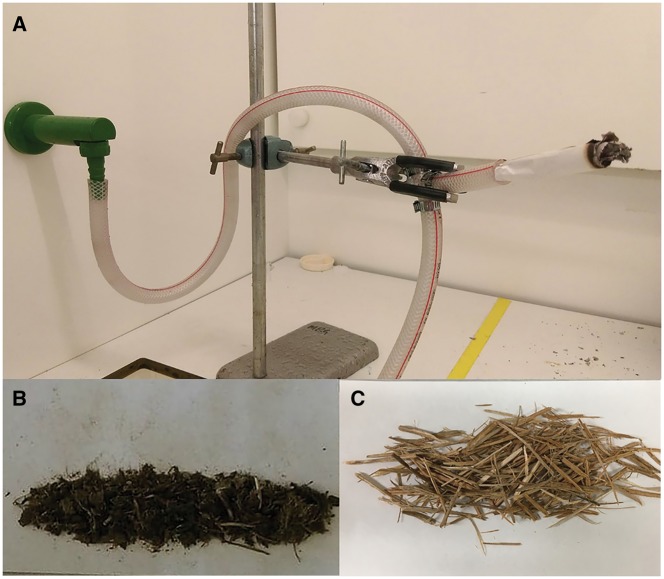
Generation of CDSE and WSE. (**A**) A water aspirator was set up to draw smoke from a burning cow dung or wood shaving roll using the vacuum created by the flow of water. (**B**) Cow dung was sun-dried, crushed into fine particles, and rolled in paper. (**C**) Wood was cut into small chips and rolled in paper.

Cigarette smoke extract (CSE) was prepared at the College of Health and Medicine, University of Tasmania, Australia. Briefly, the filter from a Marlboro cigarette butt was replaced with a sterile cotton wool filter and was smoked using a water aspirator [11]. The water aspirator consisted of a tee with hose barbs on three sides fitted with hoses. The hose-fitted tee was clamped in a stand as shown in Fig. 1A. One of the hoses from the tee fitting was connected to a tap, the second on the opposite side drained water to a sink, and the third hose at a right angle held the cigarette roll. When water was passed through the tube, a vacuum was generated by the Venturi effect, drawing smoke from a burning cigarette [18]. Here, the flow of water was maintained at the constant rate of 110 ml/s. The flow of water ensured the continual smoking of the cigarette, thereby collecting the cigarette smoke material in the cotton wool filter [11, 17]. After complete combustion of a cigarette, the cotton wool filter was removed and then placed into another cigarette from which the filter had been removed. This way, the same cotton filter was used in the smoking of three Marlboro cigarettes.

The cigarette smoke material retained in the cotton filter was quantified by measuring the weight of cotton filter before and after the combustion of the three cigarettes. The cotton filter was then vortexed in 1 ml dimethyl sulfoxide (DMSO). The solubilized smoke material was quantitated by measuring the weight of equal volumes of pure DMSO and smoke material-dissolved DMSO. The CSE was then filter-sterilized through a 0.22 μm membrane filter and the filtrate was re-quantified by weight measurement.

### Preparation of cow dung smoke extract

Cow dung, that was collected from a local farm near Hobart, Tasmania, was sun-dried for approximately 5 days and was crushed into fine particles using a mortar and pestle. Cow dung powder was rolled in a paper with a sterile cotton wool filter at one of the ends, similar to a filtered-cigarette (Fig. 1B). Four such rolls were prepared from a total of 7.5 g of cow dung fine particles, such that each roll contained 1.875 g of cow dung powder. The dung roll was then burned and smoked using the water aspirator, as described for cigarette smoking. After complete combustion of a dung roll, the cotton filter was removed and placed in another cow dung roll. In this way, four such cow dung rolls were smoked using the same cotton wool filter. Finally, the cow dung smoke extract (CDSE) was prepared by vortexing the cotton wool filter in 1 ml DMSO. The solubilized smoke material was quantitated by measuring the weight of equal volumes of pure DMSO and smoke material-dissolved DMSO. The CDSE was then filter-sterilized through a 0.22 μm membrane filter and the filtrate was re-quantified by weight measurement.

### Preparation of wood smoke extract

Fire wood was collected from a local supplier near Hobart, Tasmania. For wood smoke generation, 1.875 g of wood shavings were rolled in paper with a sterile cotton wool filter at one of the ends, similar to a filtered-cigarette (Fig. 1C). Four such rolls were prepared from a total of 7.5 g of wood shavings. Each wood shaving roll was then burned and smoked using the water aspirator, as described for cigarette smoking. After complete combustion of a wood shaving roll, the cotton filter was removed and placed into another wood shaving roll. Finally, the wood smoke extract (WSE) was prepared by vortexing the cotton wool filter in 1 ml DMSO. The solubilized smoke material was quantitated by measuring the weight of equal volumes of pure DMSO and smoke material-dissolved DMSO. The WSE was then filter-sterilized through a 0.22 μm membrane filter and the filtrate was re-quantified by weight measurement.

### Normalization of smoke extracts

To compare the effects of different smoke extracts, the prepared extracts were normalized to the same concentration and were stored at −20°C in aliquots of 100 µl until use. The normalized smoke extracts were diluted in bronchial epithelial cell growth medium (BEGM) for use in the smoke extract exposure experiments.

### *In vitro* BEAS-2B cell culture

As airway epithelial cells are the primary cells to respond to smoke, an immortalized cell line of human bronchial epithelial cells, BEAS-2B (Catalogue no 95102433, Sigma-Aldrich), was selected for this study. The BEAS-2B cells were maintained at 37°C, 5% CO_2_ in BEGM (Lonza, Basel, Switzerland) supplemented with the BulletKit (Lonza). The BEAS-2B cells were sub-cultured in T75 flasks (Corning Inc., Corning, NY, USA), and were used in experiments at passage numbers ≤15 passages. Sterile 8-well chambered glass slides (Millipore, Billerica, MA, USA) were pre-coated by incubating overnight at 4°C with 200 μl of 5% (v/v) bovine collagen I (ThermoFisher Scientific, USA), prepared in 20 mM acetic acid. The wells were rinsed twice with pre-warmed phosphate buffered saline (PBS) followed by seeding of the BEAS-2B cells at a cell density of 30 000 cells per well in 200 µl BEGM and incubated overnight at 37°C, 5% CO_2_. On the following day, the culture media was replaced with fresh BEGM and incubated at 37°C, 5% CO_2_ for 24 h before the *in vitro* smoke extract exposure experiments.

### Exposure of BEAS-2B cells to smoke extracts

Approximately 50 000–60 000 BEAS-2B cells in each well were exposed to 200 µl of BEGM containing five different concentrations of CDSE and WSE, ranging from 8.75 ng/ml to 87.5 µg/ml, for 4 h at 37°C and 5% CO_2_. Parallel exposures of BEAS-2B cells to CSE in the concentration range of 8.75 ng/ml to 87.5 µg/ml were also performed for comparison.

### Immunofluorescence

After 4 h of exposure to the smoke extracts, the media was discarded and the cells were washed twice with 200 µl PBS pre-warmed at 37°C. The cells were then fixed with 200 μl of 4% (w/v) paraformaldehyde (Sigma-Aldrich) for 20 min at room temperature. The cells were rinsed twice with 200 μl of PBS and permeabilized with 100 μl of chilled (−20°C) acetone for 10 min at room temperature. After washing the cells again twice with 200 μl of PBS, the non-specific binding sites were blocked with 200 μl of 1% (w/v) bovine serum albumin (Sigma-Adrich), prepared in PBS containing 0.1% (v/v) Tween-20 (Sigma-Aldrich), for an hour at room temperature. Cells were then incubated overnight with 100 μl of 2.5 μg/ml monoclonal antibody (mAb) against the human PAFR protein (11A4, Clone 21, Cayman Chemical Company, USA) at 4°C in the dark. The cells were rinsed 3 times with 200 μl of 0.1% (w/v) bovine serum albumin (BSA) in PBS and incubated for an hour with 100 μl of 1:100 dilutions of Alexa Fluor 594 conjugated goat anti-mouse IgG (H + L) secondary antibody (ThermoFisher Scientific, USA) at room temperature. After rinsing 3 times with 200 μl of 0.1% (w/v) BSA in PBS, the cells were stained with 200 μl of 1 μg/mL 4′, 6-diamidino-2-phenylindole dihydrochloride (DAPI) (ThermoFisher Scientific, USA) for 15 min at room temperature. Finally, the cells were washed 3 times with 200 μl of PBS, air-dried and the slides were mounted with Dako fluorescence mounting media (Agilent, USA).

### Microscopy and image analysis

Cell preparations were examined under 400× magnification using an Olympus BX50 epifluorescence microscope with NIS elements software (Nikon; Tokyo, Japan) and Cool Snap Hq2 CCD camera (Photometrics, Tucson, AZ, USA). Five images were taken per well from different points using multi-fluorescence channels designed for simultaneous detection of emission from the fluorochromes DAPI (violet excitation and blue emission, 200 ms exposure), and Alexa Fluor 594 (green excitation and red emission, 300-ms exposure). The level of cellular PAFR protein expression was quantified as a measure of total cell fluorescence intensity using the software ImageJ (NIH, USA) [19]. The cellular fluorescence was corrected against the background fluorescence using the following formula:

Total cell fluorescence = integrated density – (area of selected cell × mean fluorescence of the background).

### Quantitative real-time polymerase chain reaction analysis

The expression of PAFR was also determined at the transcriptional level using quantitative real-time polymerase chain reaction. The BEAS-2B cells were seeded into sterile clear-flat bottom 12-well plates (Corning Inc.) at a density of 2 × 10^5^ cells per well and incubated overnight at 37°C and 5% CO_2_. The next day, cells were exposed to different concentrations of CSE, CDSE, and WSE at 37°C and 5% CO_2_. After 3 h, total RNA was extracted with Tri-reagent (Sigma-Aldrich), according to the manufacturer’s instructions. It was then treated with DNase (Promega). Using a SensiFAST cDNA synthesis kit (Bioline), 490 ng of RNA was converted into first-stranded cDNA. The cDNA generated was amplified on a LightCycler 480 System (Roche) with the SensiFAST Probe No-ROX kit (Bioline) in a total volume of 20 µl. The relative fold change of mRNA expression was normalized to glyceraldehyde-3-phosphate dehydrogenase (GAPDH).

### BEAS-2B cell viability assay

The BEAS-2B cells were seeded into a sterile clear-flat bottom 96-well plate (Sigma-Aldrich) at a density of 5000 cells per well and incubated overnight at 37°C and 5% CO_2_. The cells were exposed to different concentrations of CSE, CDSE, and WSE. Alamar Blue (Life Technologies) was then added to each well at a final concentration of 10% (v/v). The absorbance readings were taken at 570 and 600 nm at 2 and 4 h post-exposure to smoke extracts at 8.75 and 87.5 µg/ml concentrations using a Spectromax Spectrophotometer Microplate Reader (Molecular Devices, USA). The percent reduction of Alamar Blue was calculated using the following formula:

$$\%\text{ Reduction of Alamar Blue Reagent }=\;\frac{\left(\text{Eoxi}600\;\times \;A570\right)\;–\;\left(\text{Eoxi}570\;\times \;A600\right)}{\left(\text{Ered}570\;\times \;C600\right)\;–\;\left(\text{Ered}600\;\times \;C570\right)}\times \;100$$

Molar extinction coefficient of oxidized Alamar Blue at 570 nm (Eoxi570) = 80 586; at 600 nm (Eoxi600) = 117 216

Absorbance of test wells at 570 nm (A570); at 600 nm (A600)

Molar extinction coefficient of reduced Alamar Blue at 570 nm (Ered570) = 155 677; at 600 nm (Ered600) = 14 652

Absorbance of negative control well (no cells) at 570 nm (C570); at 600 nm (C600)

### Statistical analysis

Data were expressed as mean ± standard error of the mean (SEM) and median (interquartile range) using the Microsoft Excel Statistics package (Microsoft Corporation, Redmond, WA, USA) and analyzed using GraphPad Prism version 5.0 for Windows (GraphPad Software, San Diego, CA, USA, www.graphpad.com). Comparisons between groups were performed using unpaired two-tailed *t*-tests with Welch’s correction and one-way analysis of variance (ANOVA) with Dunnett’s multiple comparison analysis.

## Results

### CSE, CDSE, and WSE preparation

After the combustion of three cigarettes, and 7.5 g each of cow dung powder and wood shavings, 66.52, 124.7, and 131.2 mg of smoke particles were retained in the cotton filter. The retained smoke particles were then solubilized in DMSO. The concentration of DMSO-dissolved cigarette, cow dung, and wood smoke particles were 10.85, 43.7, and 36.0 mg/ml, respectively. After filter sterilization, the final concentration of cigarette, cow dung, and wood smoke material were 8.75, 24.4, and 31.64 mg/ml, respectively. The CSE, CDSE, and WSE concentrations were all normalized to same concentration of 8.75 mg/ml and were used in subsequent exposure experiments over the range from 8.75 ng/ml to 87.5 µg/ml.

### CSE exposure increases PAFR expression on bronchial epithelial cells

**Figure 2:**
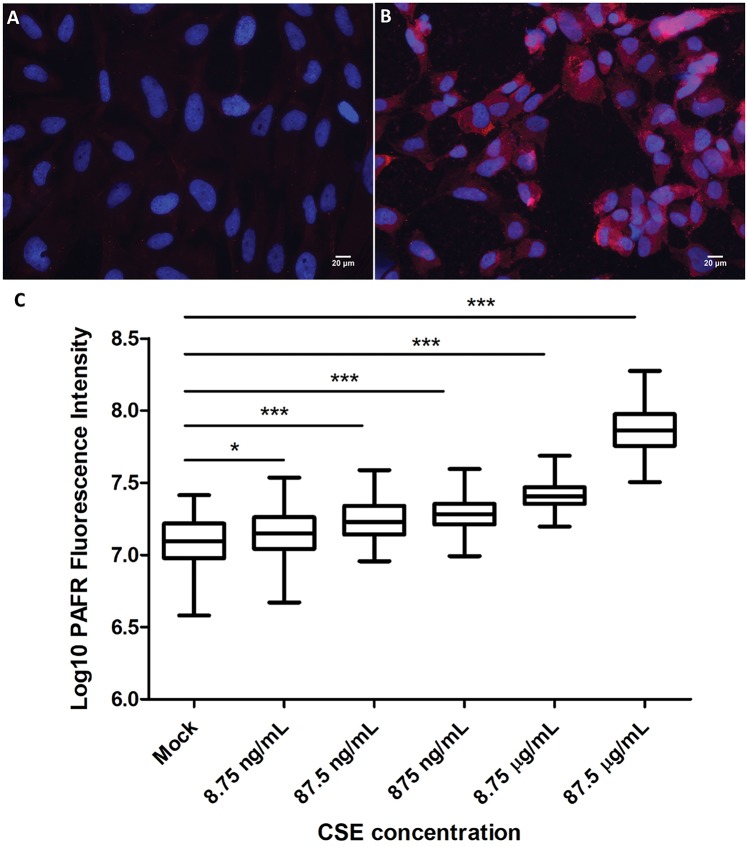
CSE exposure and PAFR expression on bronchial epithelial cells. (**A**) Mock treatment of BEAS-2B cells with 1% DMSO as a control. (**B**) BEAS-2B cells exposed to 87.5 µg/ml CSE. All immunofluorescence micrographs show BEAS-2B cells with PAFR expression (anti-PAFR monoclonal antibody; 2.5 μg/ml, red) and nuclei stained with 4′, 6-diamidino-2-phenylindole (1 μg/ml, blue). Magnification = 400×. (**C**) Response to different concentrations of CSE. PAFR expression corresponds to log_10_ of fluorescence intensity following labelling with Alexa Fluor 594 conjugate antibody targeting anti-PAFR mAb. PAFR expression was significantly increased in 8.75 ng/ml, 87.5 ng/ml, 875 ng/ml, 8.75 µg/ml and 87.5 µg/ml CSE exposed BEAS-2B cells. Data are representative of two independent experiments (*P < 0.05, ***P <* *0.0001, One-way ANOVA with Dunnett’s multiple comparison test).

PAFR expression was measured based on fluorescence intensity following labeling with Alexa Fluor 594 conjugate antibody targeting anti-PAFR mAb. Previous studies have reported that PAFR expression is upregulated in BEAS-2B cells exposed to CSE and that maximal induction occurred at 4 h of CSE exposure [11, 12]. Here, CSE exposure for 4 h significantly increased the expression of PAFR on the bronchial epithelial cells (Fig. 2A–C). We observed a dose-dependent increase in PAFR expression upon stimulation with CSE at a concentration range of 8.75 ng/ml to 87.5 µg/ml. In comparison to the mock (1% DMSO) treated control BEAS-2B cells, the mean PAFR expression was approximately 1.18, 1.39, 1.56, 2.06, and 6.10 times higher in the 8.75 ng/ml, 87.5 ng/ml, 875 ng/ml, 8.75 µg/ml, and 87.5 µg/ml CSE treated cells, respectively (Fig. 1C). The level of activity of the CSE was compared over time. There was no significant loss detected in PAFR induction following storage of CSE at −20°C freezer over a 3-month period (Fig. 5). The viability of the BEAS-2B cells exposed to 8.75 and 87.5 µg/ml of CSE for 2 and 4 h was assessed relative to mock (1% DMSO) treated control cells using the Alamar Blue assay. The mean relative viability of BEAS-2B cells treated with 8.75 µg/ml CSE for 2 and 4 h was at 106.2 ± 3.1% (SEM) and 95.8 ± 11.4%, respectively, of the control cell viability. For BEAS-2B cells treated with 87.5 µg/ml CSE for 2 and 4 h, the mean relative viability was at 100.7 ± 5.0% and 95.8 ± 11.5%, respectively.

### CDSE treatment induces PAFR expression on bronchial epithelial cells

**Figure 3:**
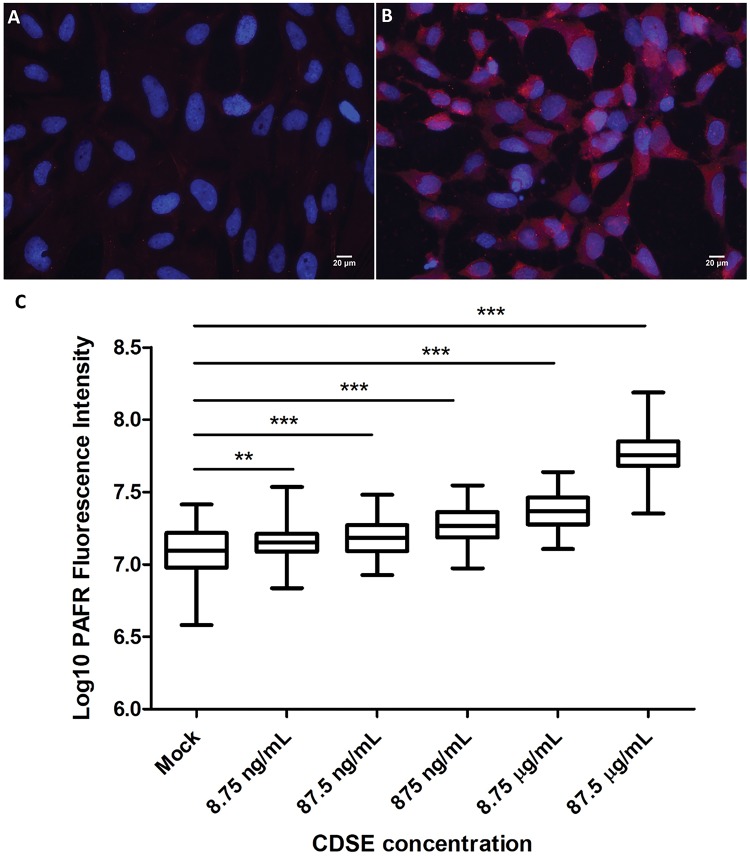
CDSE exposure and PAFR expression on bronchial epithelial cells. (**A**) Mock treatment of BEAS-2B cells with 1% DMSO as a control. (**B**) BEAS-2B cells exposed to 87.5 µg/ml CDSE. All immunofluorescence micrographs show BEAS-2B cells with PAFR expression (anti-PAFR monoclonal antibody; 2.5 μg/ml, red) and nuclei stained with 4′, 6-diamidino-2-phenylindole (1 μg/ml, blue). Magnification = 400×. (**C**) Response to different concentrations of CDSE. PAFR expression corresponds to log_10_ of fluorescence intensity following labelling with Alexa Fluor 594 conjugate antibody targeting anti-PAFR mAb. The PAFR expression was significantly increased in 8.75 ng/ml, 87.5 ng/ml, 875 ng/ml, 8.75 µg/ml and 87.5 µg/ml CDSE treated BEAS-2B cells. Data are representative of two independent experiments (**P < 0.001, ***P < 0.0001, one-way ANOVA with Dunnett’s multiple comparison test).

The expression of PAFR on BEAS-2B cells was significantly upregulated by exposure to CDSE (Fig. 3A–C). We observed a dose-dependent increase in PAFR expression upon exposure to CDSE at a concentration range of 8.75 ng/ml to 87.5 µg/ml. The mean cellular PAFR expression was approximately 1.17, 1.24, 1.51, 1.84, and 4.67 times higher than the mock (1% DMSO) treated control BEAS-2B cells in the 8.75 ng/ml, 87.5 ng/ml, 875 ng/ml, 8.75 µg/ml, and 87.5 µg/ml CDSE-stimulated cells, respectively (Fig. 3C). The level of induction of PAFR expression due to CDSE exposure was comparable in experiments conducted 3 months apart (Fig. 5). From the Alamar Blue assay, the mean relative viability of BEAS-2B cells exposed to 8.75 µg/ml CDSE for 2 and 4 h was at 97.4 ± 6.1% and 97.2 ± 13.9%, respectively, of the control cell viability. For BEAS-2B cells treated with 87.5 µg/ml CDSE for 2 and 4 h, the mean relative viability was at 102.2 ± 7.9% and 90.8 ± 10.7%, respectively.

### WSE exposure upregulates PAFR expression on bronchial epithelial cells

**Figure 4:**
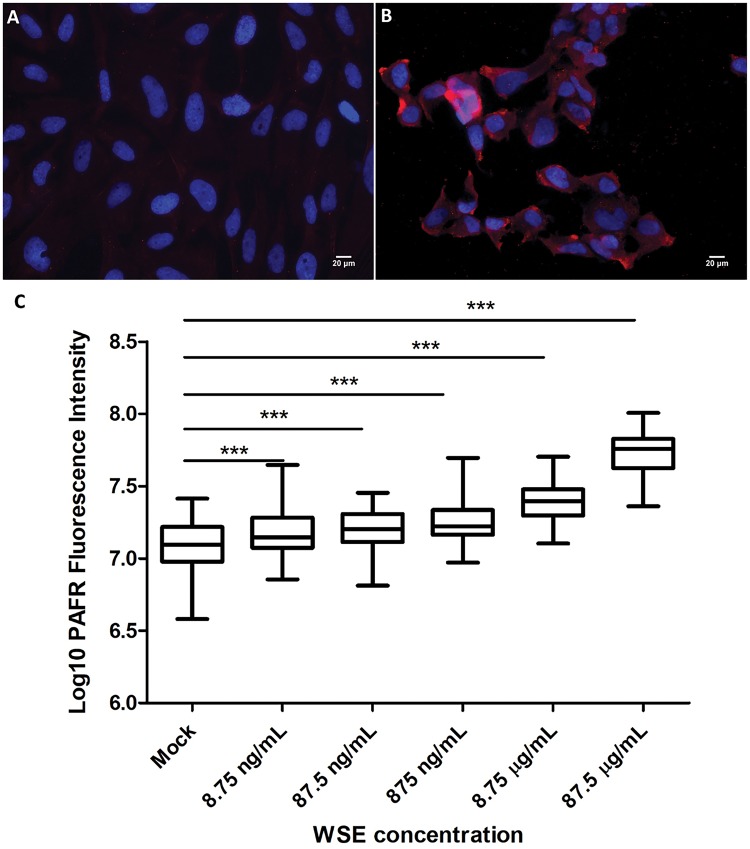
Wood smoke extract (WSE) exposure and PAFR expression on bronchial epithelial cells. (**A**) Mock treatment of BEAS-2B cells with 1% DMSO as a control. (**B**) BEAS-2B cells exposed to 87.5 µg/ml WSE. All immunofluorescence micrographs show BEAS-2B cells with PAFR expression (anti-PAFR monoclonal antibody; 2.5 μg/ml, red) and nuclei stained with 4′, 6-diamidino-2-phenylindole (1 μg/ml, blue). Magnification = 400×. (**C**) Response to different concentrations of WSE. PAFR expression corresponds to log_10_ of fluorescence intensity following labelling with Alexa Fluor 594 conjugate antibody targeting anti-PAFR mAb. The PAFR expression was significantly increased in 8.75 ng/ml, 87.5 ng/ml, 875 ng/ml, 8.75 µg/ml and 87.5 µg/ml WSE exposed BEAS-2B cells. Data are representative of two independent experiments (***P < 0.0001, one-way ANOVA with Dunnett’s multiple comparison test).

**Figure 5:**
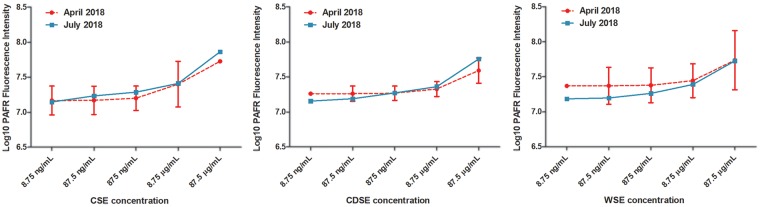
Comparison of effects of smoke extracts on PAFR expression in experiments conducted 3 months apart. All of the smoke extracts, CSE, CDSE, and WSE, were run in the concentration range from 8.75 ng/ml to 87.5 µg/ml in the July 2018 experiment. The CSE extract was run in the same concentration range from 8.75 ng/ml to 87.5 µg/ml in the April 2018 experiment. For the April 2018 experiments, the CDSE and WSE data were interpolated to the 8.75 ng/ml to 87.5 µg/ml concentration range using non-linear least squares regression. The levels of PAFR expression in the July 2018 experiments with the CSE, CDSE, and WSE samples were within 95% CI of the levels obtained for the April 2018 experiments. Therefore, no significant decay in the PAFR inducing activity of the smoke extracts was detected following storage at −20°C over a 3-month period.

Wood smoke extract exposure was also associated with an induction of PAFR expression on the bronchial epithelial cells (Fig. 4A–C). Treatment with WSE for 4 h resulted in a concentration-dependent increase in the expression of PAFR on BEAS-2B cells. Compared to the 1% DMSO-treated control cells, the mean cellular PAFR expression was approximately 1.28, 1.27, 1.50, 1.99, and 4.34 times higher in 8.75 ng/ml, 87.5 ng/ml, 875 ng/ml, 8.75 µg/ml, and 87.5 µg/ml WSE exposed BEAS-2B cells, respectively (Fig. 4C). The PAFR inducing activity of WSE was similar in experiments conducted 3 months apart (Fig. 5). From the Alamar Blue assay, the mean relative viability of BEAS-2B cells exposed to 8.75 µg/ml WSE for 2 and 4 h was at 101.5 ± 9.2% and 83.8 ± 8.2%, respectively, of the control cell viability. For BEAS-2B cells treated with 87.5 µg/ml WSE for 2 and 4 h, the mean relative viability was at 93.8 ± 7.4% and 79.2 ± 8.1%, respectively.

### Transcriptional response of BEAS-2B to CSE, CDSE, and WSE

To examine the expression of PAFR at the transcriptional level, the relative PAFR mRNA expression, normalized to glyceraldehyde-3-phosphate (GAPDH), was measured post-exposure to the smoke extracts at 8.75 and 87.5 µg/ml concentrations. Compared to mock (1% DMSO) treated controls, the transcriptional level of PAFR was increased 2.45-, 3.37-, and 2.65-fold after exposure to CSE, CDSE, and WSE, respectively, at 8.75 µg/ml (Table 1). The mRNA levels of PAFR were 3.19-, 4.17-, and 3.38-fold higher in BEAS-2B cells exposed to 87.5 µg/ml concentrations of CSE, CDSE, and WSE, respectively.

**Table 1:** The smoke extracts CSE, CDSE, and WSE induce PAFR, IL-1β, IL-6, and IL-8 mRNA expression in BEAS-2B cells

Marker	CSE		CDSE		WSE	
	A	B	A	B	A	B
PAFR	2.45 ± 0.08***	3.19 ± 0.69	3.37 ± 0.18***	4.17 ± 1.13	2.65 ± 0.25**	3.38 ± 0.5*
IL-1β	5.56 ± 0.65**	8.06 ± 2.95	8.26 ± 0.27***	14.32 ± 4.73	9.6 ± 1.24**	6.69 ± 0.45**
IL-6	7.15 ± 0.58**	13.83 ± 4.95	12.39 ± 0.4***	12.02 ± 1.57**	10.93 ± 1.05**	13.42 ± 5.74
IL-8	10.86 ± 1.61**	11.44 ± 4.42	11.64 ± 1.0**	20.25 ± 2.43**	10.62 ± 0.6***	8.67 ± 1.81*

Relative fold change in mRNA of PAFR and inflammatory cytokines normalized with GAPDH among BEAS-2B cells exposed to CSE, CDSE, and WSE for 3 h at concentrations (A) 8.75 µg/ml and (B) 87.5 µg/ml (*P < 0.05, **P < 0.001, ***P < 0.0001, unpaired two-tailed *t*-test with Welch’s correction. The data are presented as the mean of the observed fold change ± SEM, *n* = 4 per group.

Previous studies have reported a respiratory inflammatory response to cigarette, animal dung, and wood smoke exposure [9, 20–23]. To investigate the inflammatory response *in vitro*, the BEAS-2B cells were exposed to 8.75 and 87.5 µg/ml concentrations of CSE, CDSE, and WSE for 3 h and mRNA expression was measured. The mRNA levels for pro-inflammatory cytokines, interleukin-1 beta (IL-1β), IL-6, and IL-8 were increased by 5.56-, 7.15-, and 10.86-fold, respectively, post-exposure to 8.75 µg/ml CSE (Table 1). Exposure of BEAS-2B cells to 87.5 µg/ml CSE resulted in an 8.1-, 13.8-, and 11.4-fold increase in mRNA levels of the inflammatory mediators, IL-1β, IL-6, and IL-8. A similar increase in inflammatory mediators IL-1β, IL-6, and IL-8 was observed upon exposure of BEAS-2B cells to 8.75 and 87.5 µg/ml concentrations of CDSE and WSE (Table 1).

## Discussion

Nearly 4.3 million people die every year from illnesses attributable to the inhalation of biomass smoke [1]. Among these deaths, 22% are due to COPD and 12% due to pneumonia [1]. Biomass fuels (wood, animal dung, and crop residues) are the major source of domestic energy for cooking and household heating, especially in developing countries. Emissions from biomass contain a multitude of pollutants that adversely affect human health, such as suspended particulate matter, methane, free radicals, aldehydes, toxic gases like carbon monoxide and nitrogen oxides, and polycyclic aromatic hydrocarbons like benzo[*a*]pyrene and anthracene [24]. Furthermore, animal dung combustion produces more toxic byproducts, including particulates (23% more PM_2.5_ per kilogram of sample), reactive oxygen species, and microbial products, compared to wood smoke [10, 25]. Several epidemiological studies have correlated biomass smoke exposure with the risk of development of lung diseases, including COPD, lung cancer, and airway infections [3–7]. However, there are only a limited number of studies that have explored mechanisms in biomass smoke induced-pulmonary inflammation and susceptibility to respiratory infections [26]. This knowledge gap is in part due to the lack of a standardized low-cost technique for the generation of biomass smoke in the laboratory.

In work by McCarthy and colleagues, biomass smoke from the combustion of horse dung was pumped into a chamber in which human small airway epithelial cells were exposed [9]. While this method delivered smoke to the epithelial cells, it involved immediate use of the smoke generated and did not allow for storage of batches of biomass smoke for subsequent re-use [9]. It also required the employment of a cigarette smoking machine (Baumgartner-Jaeger CSM2072i) to generate the smoke. In a study by Li and co-workers, biomass smoke from the combustion of rice chaff was bubbled through the cell culture growth medium, Dulbecco’s modified Eagle’s medium [27]. Again this method generated biomass smoke but the extracts could not be quantified in terms of mass per volume due to the presence of multiple nutrient elements in the growth medium. Furthermore, many components of biomass smoke are not directly soluble in aqueous solutions such as growth medium, and as such will not be retained when the smoke is bubbled through the medium [27]. In work by Huang and co-investigators, wood smoke from the burning of Chinese fir was collected directly onto a glass filter with a 1.6-µm pore size [28]. This method collected wood smoke particles as intended but it is likely that many of the smaller components of the smoke, such as volatile organic compounds, from the combustion of the wood would have passed through rather than have been captured on the glass filter.

In our method, the smoke material that was collected in the cotton wool was first incubated in the solvent DMSO, which dissolves both polar and nonpolar compounds, overnight before the filtration step to maximize solubilization of the components. Furthermore, most of the smoke material in the DMSO was retained after the filter sterilization step based on both weight and absorbance measurements. By including quantification measurement at several of the preparation steps, we were able to determine the concentration of smoke-derived material in milligram per milliliter in each of the smoke extracts. In addition, we were able to generate batches of smoke extracts that could be preserved indefinitely and used in multiple exposure experiments, minimizing inter-assay variation. And importantly, our protocol does not require the purchase of expensive equipment and therefore, is suitable for use in resource-limited situations.

To test our biomass smoke extracts, we compared their effect on the expression of PAFR on the human bronchial epithelial cells. PAFR is a G-protein-coupled seven transmembrane domain receptor, involved in various leukocyte functions, platelet aggregation, and inflammation [29]. Previous studies have shown that PAFR expression is upregulated in response to a variety of insults including cigarette smoke, e-cigarette vapor, urban particulate matter, and welding fumes [30–33]. In terms of infection, PAFR is utilized by major respiratory bacterial pathogens including non-typeable *Haemophilus influenzae*, *Streptococcus pneumoniae*, and *Pseudomonas aeruginosa* as a surface receptor for adhesion of airway epithelial cells. These species express a common adhesin, known as phosphorylcholine (ChoP), in their cell wall that recognizes and binds host cell PAFR enabling establishment of infection of the respiratory tract [11, 34, 35].

We determined that PAFR expression is increased in bronchial epithelial cells following exposure to CDSE and WSE in a dose-dependent manner at both the protein and mRNA levels (Figs. 2–4, Table 1). In addition, the PAFR-inducing activity of the smoke extracts was comparable in experiments conducted 3 months apart (Fig. 5). Therefore, the activity of the smoke extracts was preserved during storage at −20°C in DMSO for at least the 3-month period tested. The upregulation of PAFR may represent a molecular mechanism through which these biomass smoke types could increase susceptibility to lung diseases including airway infections. Furthermore, we detected increased expression of pro-inflammatory mediators IL-1β, IL-6, and IL-8 following exposure of BEAS-2B cells to our CSE, CDSE, and WSE preparations in accordance with earlier studies on the effect of smoke on respiratory cells (Table 1). Therefore, the ability to produce CDSE and WSE in a usable form, by applying a simple and cost-effective water aspirator-based method, will enable further research on their mechanistic role in the inflammatory response and pathogenesis of respiratory disease including COPD. In addition, it will facilitate the discovery of novel therapeutic compounds that reduce the effects of biomass smoke on host cells and tissues of the respiratory system.
